# Pay Hospitals

**Published:** 1891-01-03

**Authors:** 


### PAY HOSPITALS.

**Hampstead Home Hospital.**—This excellent little hospital has been steadily increasing its work, and yearly showing a greater appreciation evinced towards it by the increased applications for private nurses. The accommodation in the hospital now comprises a female ward, containing eight beds, a male ward with three beds, and a children's ward with seven cots, all of whom pay some small sum towards the expenses of their residence in the hospital. The patients increase in numbers yearly; 118 cases, including children, have been under treatment, and an out-patients' department may be considered as commenced. The principles on which this hospital works are admirably adapted to meet the needs of the poorer middle classes. Without lowering their self-esteem, or pauperising them by entirely gratis attendance, the advantages of hospital residence are offered them for a small weekly sum, a boon to a large section of the community, who would infinitely prefer to pay for what they receive as far as their means will permit. As, however, the amount paid is inadequate to defray the cost of maintenance, we hope that the able secretary, Mr. R. A. Crothwaite, will receive many contributions towards its support, which should be addressed to him at the Institute, Hill Road, N.W.

